# Diabetic dyslipidemia and its predictors among people with diabetes in Ethiopia: systematic review and meta-analysis

**DOI:** 10.1186/s13643-024-02593-2

**Published:** 2024-07-20

**Authors:** Abere Woretaw Azagew, Hailemichael Kindie Abate, Chilot Kassa Mekonnen, Habtamu Sewunet Mekonnen, Zewdu Baye Tezera, Gashaw Jember

**Affiliations:** 1 Department of Medical Nursing, School of Nursing, College of Medicine and Health Sciences, University of Gondar, Gondar, Ethiopia; 2 Department of Comprehensive Nursing, School of Nursing, College of Medicine and Health Sciences, University of Gondar, Gondar, Ethiopia; 3 Department of Physiotherapy, School of Medicine, College of Medicine and Health Sciences, University of Gondar, Gondar, Ethiopia

**Keywords:** Diabetes mellitus, Dyslipidemia, Lipid profile abnormality, Ethiopia

## Abstract

**Background:**

Dyslipidemia is an imbalance of lipid profiles. It increases the chance of clogged arteries and may cause heart attacks, strokes, and other circulatory disorders. Dyslipidemia affects the general population, but its severity is higher in diabetic populations. As a result, the chance of dyslipidemia-associated morbidity and mortality is highest in diabetic patients. In Ethiopia, around 2 to 6.5% of the population live with diabetes, but their lipid profiles are inconsistent across the studies. Therefore, this study aimed to estimate the pooled prevalence of diabetic dyslipidemia and its predictors among people with diabetes in Ethiopia.

**Method:**

A systematic review and meta-analysis was conducted. The searches were carried out in MEDLINE via PubMed and OVID, EBSCO, Embase, and other supplementary gateways such as Google and Google Scholar, for articles published up to June 2023. The articles were searched and screened by title (ti), abstract (ab), and full text (ft). The quality of the eligible studies was assessed by the Newcastle–Ottawa scale. The heterogeneity was detected by the Cochrane Q statistic test and the I-squared (*I*^2^) test. Then subgroup analysis and meta-regression analysis were used to identify the source of the variations. A random or fixed-effect meta-analysis model was used to estimate the overall pooled prevalence and average effects. The publication bias was assessed by the funnel plot asymmetry test and/or Begg and Mazumdar’s test for rank correlation (*p*-value < 0.05). The protocol has been registered in an international database, the prospective register of systematic reviews (PROSPERO), with reference number CRD42023441572.

**Result:**

A total of 14 articles with 3662 participants were included in this review. The pooled prevalence of diabetic dyslipidemia in Ethiopia was found to be 65.7% (95% confidence interval (CI): 57.5, 73.9), *I*^2^ = 97%, and *p*-value < 0.001. The overall prevalence of triglycerides (TG) and high-density lipoprotein cholesterol (HDL-c) were found to be 51.8% (95% *CI*: 45.1, 58.6) and 44.2% (95% *CI*: 32.8, 55.7), respectively, among lipid profiles. In meta-regression analysis, the sample size (*p* value = 0.01) is the covariate for the variation of the included studies. Being female (adjusted odds ratio (AOR): 3.9, 95% *CI*: 1.5, 10.1), physical inactivity (*AOR*: 2.6, 95% *CI*: 1.5, 4.3), and uncontrolled blood glucose (*AOR*: 4.2, 95% *CI*: 1.9, 9.4) were found to be the determinants of dyslipidemia among diabetic patients.

**Conclusion:**

This review revealed that the prevalence of diabetic dyslipidemia is high among people with diabetes in Ethiopia. Being female, having physical inactivity, and having uncontrolled blood glucose were found to be predictors of dyslipidemia among people with diabetes. Therefore, regular screening of lipid profiles and the provision of lipid-lowering agents should be strengthened to reduce life-threatening cardiovascular complications. Furthermore, interventions based on lifestyle modifications, such as regular physical activity and adequate blood glucose control, need to be encouraged.

**Supplementary Information:**

The online version contains supplementary material available at 10.1186/s13643-024-02593-2.

## Background

Diabetes mellitus (DM) is the leading public health challenge worldwide [1]. Globally, around 537 million people have DM. Over three in four adults with diabetes live in low- and middle-income countries (LMICs). It is one of the top ten leading causes of death globally [2] and is responsible for the direct cause of 1.5 million deaths [3]. Similarly, diabetes is one of the top nine causes of death in LMICs [4]. The prevalence of DM in Ethiopia has been reported to range from 2 to 6.5% [5].

Dyslipidemia is a lipoprotein metabolism disorder, including lipoprotein overproduction or deficiency [6, 7]. It is characterized by elevated fasting and postprandial triglycerides (TG), low in high-density lipoprotein-cholesterol (HDL-C), elevated low-density lipoprotein cholesterol (LDL-C), and the predominance of low-density lipoprotein (LDL) particles [8]. The causes of dyslipidemia may be primary (genetic) or secondary, which are caused by lifestyle or other factors. It affects the general population, but its prevalence is highest in diabetic patients [9]. The mechanism for the development of dyslipidemia is not clearly understood in diabetics, but it is associated with insulin resistance, elevated blood glucose levels, excessive production of rich lipoproteins from the liver [10], and problems with lipid enzymes. About 61% of diabetic patients have at least one or more elevated liver enzymes [11]. A high-fat and high-calorie diet can cause dyslipidemia and lead to endothelial dysfunction [12]. Dyslipidemia clogs arteries, which contributes to the development of atherosclerosis and cardiovascular disease. These result in coronary artery diseases, peripheral vascular diseases, heart attacks, stroke, hypertension, heart failure, renal disease, and others [13, 14]. In order to overcome the negative effects of dyslipidemia, intervention strategies including regular follow-up care, health education on lifestyle modifications, and providing lipid-lowering agents have been implemented [15–17]. In Ethiopia, studies have reported on the prevalence of dyslipidemia among people with diabetes, but their findings are inconsistent across studies. Therefore, this review aimed to estimate the pooled prevalence of dyslipidemia and identify its predictors among people with diabetes in Ethiopia.

## Methods

### Study protocol registration and reporting

The protocol for this review has been registered on PROSPERO with reference number CRD42023441572. The report of this review followed the systematic review and meta-analysis (PRISMA) guideline checklist [18] (Additional File 1).

### Searching strategies and selections

The searches were carried out in MEDLINE via PubMed and OVID, EBSCO, Embase, and other supplementary gateways such as Google and Google Scholar. The Boolean operators of OR and AND were used to combine the search terms. Articles were searched by title (ti), abstract (ab), and/or full text (ft). The search string is stated as dyslipidemia OR “abnormal lipid profile*” OR hyperlipidemia AND “diabetes mellitus” OR DM OR “Type 2 diabetes mellitus” OR T2DM OR “Type 1 diabetes mellitus” OR T1DM AND Ethiopia. All search results were exported to Rayyan software to screen and de-duplicate the articles. Two collaborators (Z. B. T. and G. J.) were invited and screened articles independently based on the eligibility criteria. Then, the disagreements were resolved through discussion. EndNote reference management software version 7 was used to cite the references. The searches included articles published up to June 2023. The search was carried out from June 1 to 30, 2023.

### Inclusion and exclusion criteria

Studies were eligible for inclusion in the review if they reported on the following: (1) adult (age ≥ 18 years), diabetic (type 1 and/or type 2 diabetic), and population groups (2) were observational in design (i.e., cross-sectional, cohort, and case control); (3) reported the prevalence, proportion, or incidence of dyslipidemia; (4) were conducted in Ethiopia; (5) were published or unpublished; and (6) took place in primary, general, and/or tertiary hospitals and were included in the study. On the contrary, articles with abstracts only and conference papers were excluded. Moreover, articles with narrative reviews, systematic reviews, and meta-analyses were also excluded.

### Outcome measurement

Dyslipidemia was defined as *TC* ≥ 200 mg/dl, or *TG* ≥ 150 mg/dl, or *LDL-C* ≥ 130 mg/dl, or *HDL-C* < 40 mg/dl (for men) and *HDL-C* < 50 (for women) [12]. Diabetes was defined as fasting blood glucose ≥ 126 mg/dl, *HbA1c* ≥ 7%, or patients on antidiabetic medication treatments [19].

### Quality assessment

The quality of the included studies were assessed by a modified Newcastle–Ottawa quality assessment scale (NOS) adapted from cross-sectional [20] and case–control studies [21]. The tool used a “star scoring system” based on three parameters. These were study selection, comparability of the group, and ascertainment of the exposure and outcome status. For cross-sectional studies, a maximum of 9 stars could be allocated, and for case–control studies, a maximum of 10 stars [22]. For the current review, a study of any design that received a rating of 7 or more stars was considered to be of high methodological quality [21]. Two reviewers independently reviewed the quality of the included studies, and any discrepancies between reviewers were resolved by discussion.

### Data extraction

The data were extracted using the Microsoft Word data extraction format. The format was developed by involving all the reviewers. The data extraction format was piloted among two eligible studies before extracting the data. Then, further modifications were made accordingly. The extracted data include author(s), year of publications, region, study design, population, sample size, data collection procedure, prevalence, and funding source (Additional File 2). Furthermore, to assess the predictors of diabetic dyslipidemia, the effect estimate so-called odds ratio was used. This was taken from the reports of the previous studies or calculated from their cross-tabs [23]. Two reviews (Z. B. T. and G. J.) independently retrieved the data from the studies. The disagreements between the reviewers were resolved by the discussion or involvement of the third reviewer.

### Data analysis

The retrieved data were exported into Stata version 14 for analysis. A random and/or fixed-effect meta-analysis model was used to estimate the pooled prevalence and average effects on dyslipidemia based on the presence of heterogeneity. The presence of heterogeneity was detected by a nonparametric statistical test called the Cochrane Q test, whereas the proportion of heterogeneity was estimated using I squared (*I*^2^) statistics. The *I*^2^ test statistics of 0.0 to 30%, 30 to 60%, and 60 to 100% were considered minimal, moderate, and substantial heterogeneity [24, 25], respectively. To see the level of variation across the studies, subgroup analyses by region of the study were computed. Furthermore, a random-effect meta-regression analysis was carried out to identify the covariates of the variations. As a result, publication years and sample size were used for the regression analysis. The publication bias was detected by visual inspection of the funnel plot and/or Begg’s or Egger’s linear regression test (*p*-value < 0.05).

## Results

### Study selection and characteristics

**Fig. 1:**
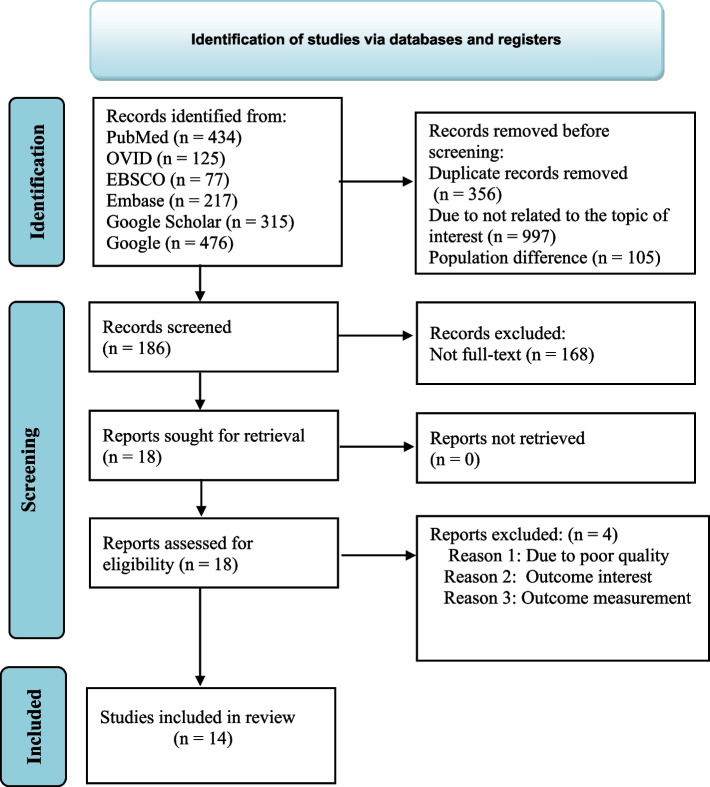
PRISMA flow diagram for the flow of information through the phases of the review

The search retrieved 1644 original research articles. From this, 356, 997, and 105 articles were removed due to duplication, background articles, and population differences, respectively. Then, after, 186 articles were retrieved, and 168 articles were removed, of which 10 were removed because not full text (abstracts only) and 158 were not related to the topic of interest. About 18 full-text articles were accessed for eligibility, of which four articles were excluded because of poor quality after NOS assessment, reporting without the outcome of interest, and outcome measurement (Fig. 1). Finally, 14 articles with 3662 diabetic patients were included and retrieved for this review. From studies, five were in Ahmara [26–30], four in SNNP [31–34], three in Oromia [35–37], one in Tigry [38], and one in Addis Ababa [39]. Thirteen were cross-sectional in design, and the remaining was case control. The publication years of the included studies were between 2017 and 2022. The data collection methods of the majority of the included studies were interviewer-administered surveys. The overall quality appraisal of the included studies was ≥ 7 (Table 1).

**Table 1:** Characteristics of the included studies

**Author**	**Year**	**Population**	**Region**	**Design**	**Study setting**	**Data collection**	**Treatment**	**Funding**	**Quality**
Abdissa D. & Hirpa [35]	2022	DM	Oromia	Cross-sectional	Primary, general, and referral hospitals	IAS	Oral agents and insulin	NR	7.5
Bekele S. et al. [31]	2017	DM	SNNPR	Cross-sectional	General hospital	IAS	Not explained	NR	8
Haile K. & Timerga A. [36]	2020	T2DM	Oromia	Cross-sectional	Tertiary hospital	IAS	Not explained	JU	9
Kebede W. M. et al. [26]	2021	T2DM	Amhara	Cross-sectional	Tertiary hospital	IAS	Not explained	Nil	8.5
Woyesa S. et al. [37]	2021	DM	Oromia	Cross-sectional	Tertiary hospital	IAS	Insulin and oral agents	JU	7
Wuhib M. et al. [28]	2021	DM	Amhara	Cross-sectional	Tertiary hospital	IAS and lab test	Not explained	NR	9
Fikremariam T. & Reddy P. P. [29]	2020	DM	Amhara	Cross-sectional	Tertiary hospital	IAS and lab test	Not explained	BDR	7.5
Biadgo B. et al. [27]	2018	T2DM	Amhara	Cross-sectional	Tertiary hospital	IAS and lab test	Not explained	Nil	8.5
Birarra M. K. & Gelaye D. A. [30]	2018	T2DM	Amhara	Cross-sectional	Tertiary hospital	IAS and lab test	Not explained	NR	8
Woyesa S. B. et al. [32]	2017	T2DM	SNNP	Cross-sectional	Tertiary hospital	IAS and lab test	Not explained	JU	8.5
Gebremeskel G. G. et al. [38]	2019	T2DM	Tigray	Cross-sectional	Tertiary hospital	IAS and record review	Insulin and oral agents	Nil	7
Abebe G. et al. [33]	2022	T2DM	SNNPR	Case control	General hospital	IAS and record review	Not explained	AMU	9
Wube B. T. et al. [34]	2020	T2DM	SNNPR	Cross-sectional	Tertiary hospital	IAS	Not explained	NR	8.5
Birle M. et al. [39]	2019	T2DM	AA	Cross-sectional	Tertiary hospital	IAS and lab test	Not explained	AMU	7.5

*AA* Addis Ababa, *AMU* Arba Minch University, *BDU* Bahir Dar University, *DM* Diabetes mellitus, Jimma University, *IAS* Interviewer-administered survey, *T2DM* Type 2 diabetes mellitus, *NR* Not reported, *SNNPR* Southern Nation, Nationalities, and People of Region

The prevalence of diabetic dyslipidemia ranges from 37.5% [37] to 91.1% [34]. The predominant lipid abnormality was elevated triglycerides. Regarding factors, two articles for sex [35, 37], two articles for physical activity [29, 36], and two articles for blood glucose control status [29, 36] were used (Table 2).

**Table 2:** Diabetes and lipid profiles related characteristics among people with diabetes in Ethiopia

**Author**	**Sample**	**Proportion of T1DM**	**Proportion of T2DM**	**Dyslipidemia (%)**	**Lipid profiles (%)**				**Variables**	**OR**
					**TC**	**LDLC**	**HDLC**	**TG**		
Abdissa D. & Hirpa [35]	390	28.50%	71.5%	81.5	29.2	16.7	47.9	63.3	Sex (female)	2.9
Bekele S. et al. [31]	224	-Not explained	Not explained	65.6	23.7	43.8	41.9	40.6	-	-
Haile K. & Timerga A. [36]	248	-	100%	68.1	13.7	28.6	50.8	48	Physical inactivity	2.5
									Uncontrolled blood glucose	3.1
Kebede W. M. et al. [26]	347	-	100%	59	36.1	59	83.8	42.2		
Woyesa S. et al. [37]	48	-Not explained	Not explained	37.5	21.8	32.3	24	26	Sex (female)	9.2
Wuhib M. et al. [28]	250	50%	50%	68	26.4	39.2	37.6	33.6	-	-
Fikremariam T. & Reddy P. P. [29]	112	Not explained	Not explained	69.6	25.9	18.8	69.6	43.8	Physical inactivity	2.8
									Uncontrolled blood glucose	7.3
Biadgo B. et al.[27]	159	-	100%	56.6	64.2	57.9	31.5	62.3	-	-
Birarra M. K. & Gelaye D. A. [30]	256	-	100%	68.8	-	-	33.6	67.2	-	-
Woyesa S. B. et al. [32]	319	-	100%	70.4	-	-	39.2	70.4	-	-
Gebremeskel G. G. et al. [38]	419	-	100%	45.1	-	-	34.4	45.1	-	-
Abebe G. et al. [33]	204	-	100%	82.18	72.55	-	11.76	59.3	-	-
Wube B. T. et al. [34]	314	-	100%	91.1	32.8	14.3	60.8	70.4	-	-
Birle M. et al. [39]	372	-	100%	51.34	-	-	51.08	47.85	-	-

*CI* Confidence interval, *HDLC* HDL-C-high-density lipoprotein cholesterol, *LDLC* Low-density lipoprotein cholesterol, *OR* Odds ratio, *TC* Total cholesterol, *TG* Triglycerides

### Prevalence of diabetic dyslipidemia

**Fig. 2:**
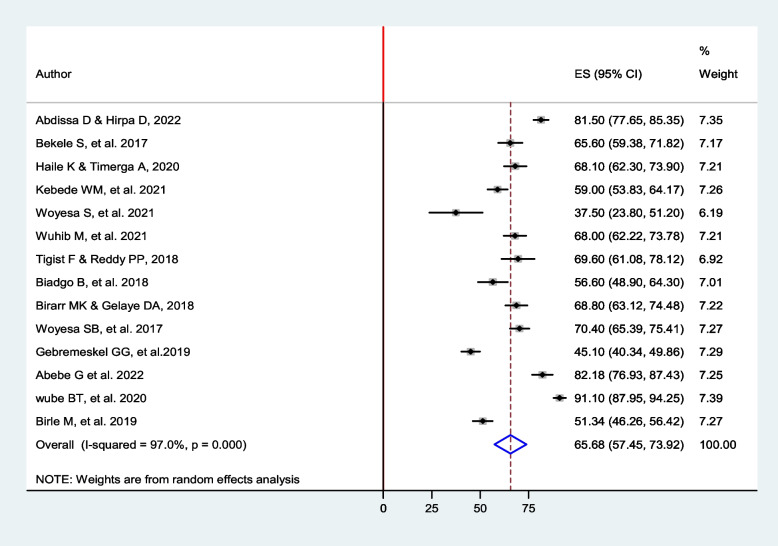
Forest plot shows the pooled prevalence of dyslipidemia among diabetic patients in Ethiopia

The pooled prevalence of dyslipidemia among people with diabetes in Ethiopia was found to be 65.68% (95% *CI*: 57.5, 73.9), *I*^2^ = 97%, *p*-value < 0.001) using a random-effect meta-analysis model (Fig. 2). Regarding the lipid profiles, the pooled prevalence of TG and HDL-C was 51.8% (95% *CI*: 45.1, 58.6) and 44.2% (95% *CI*: 32.8, 55.7), respectively (Table 3).

**Table 3:** Lipid profiles among diabetic patients

Lipid profiles	Prevalence with 95% *CI*	I-square (%)	Cochrane (*p*-value)	Egger’s test (*p*-value)	Model
TC	34.7% (23.3, 46.0)	97.3	*p* < 0.001	0.39	Random effect meta-analysis
TG	51.8% (45.1, 58.6)	94.3	*p* < 0.001	0.09	
LDL-c	34.4% (22.3, 46.6)	97.5	*p* < 0.001	0.11	
HDL-c	44.2% (32.8, 55.7)	98.2	*p* < 0.001	0.85	

*CI* Confidence interval, *HDL-c* High-density lipoprotein cholesterol, *LDL-c* Low-density lipoprotein cholesterol, *TC* Total cholesterol, *TG* Triglyceride

### Heterogeneity test

As shown in the forest plot (Fig. 2), the proportion of variation was 97% and the Cochrane Q statistic *p*-value < 0.001, indicating there is considerable variation across the studies. Therefore, further subgroup analysis and random-effect meta-regression analysis were computed.

### Subgroup analysis

**Fig. 3:**
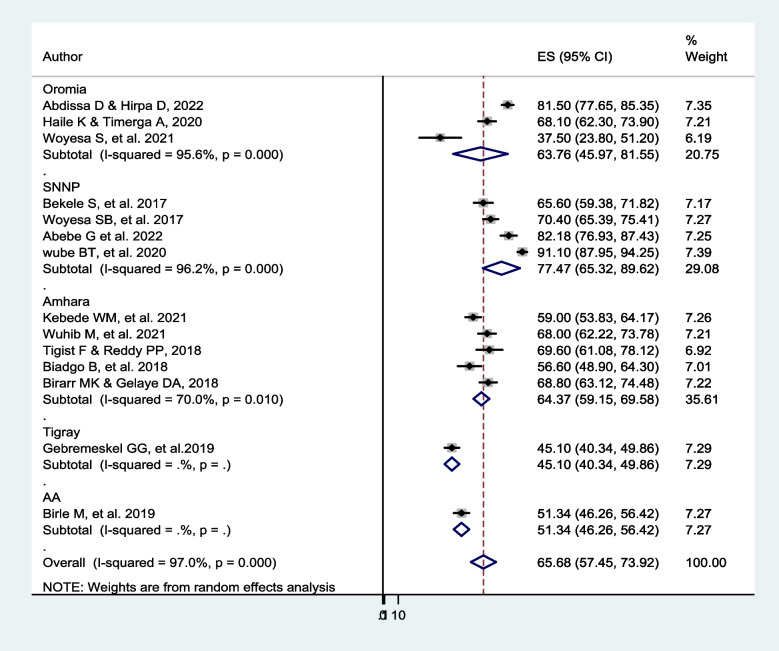
The subgroup analysis of the included studies

The subgroup analysis was computed by the region of the studies. From the included studies, the pooled prevalence of dyslipidemia among people with diabetes was 77.5% (95% *CI*: 65.3, 89.6), *I*^2^ = 96.2%, *p* < 0.001 in SNNP, 64.4% (95% *CI*: 59.2, 69.6), *I*^*2*^ = 70%, *p* = 0.01 in Amhara, and 63.8% (95% *CI*: 46.0, 81.6), *I*^2^ = 95.6%, *p* = 0.001 in the Oromia region using a random-effect meta-analysis model (Fig. 3).

### Meta-regression

To identify the factors attributed to the variations across the included studies, sample size and publication years were used. In this random effect meta-regression analysis, the sample size is found to be the covariate, possibly the source of variation or heterogeneity across the included studies (*p*-value = 0.01) (Table 4).

**Table 4:** Meta-regression analysis of studies on diabetic dyslipidemia

Heterogeneity	Coefficient	Std. err	*p*-value	95% *CI*	Model
Sample size	4.3	0.95	0.01	2.2, 6.4	Random-effect meta-regression analysis
Publication year	− 0.14	0.27	0.68	− 4.7, 1.7	

*CI* Confidence interval, *Std err* Standard error

### Publication bias

**Fig. 4:**
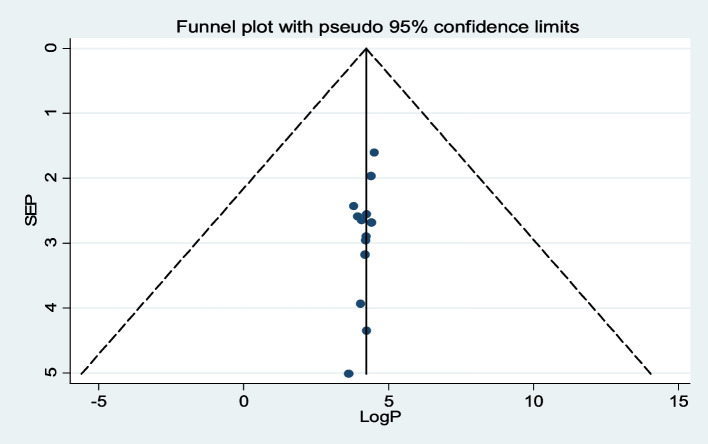
Funnel plot shows the distribution of included articles

The publication bias was detected by visual inspection of the funnel plot, indicating a symmetrical distribution of articles, and Egger’s linear regression test *p*-value was 0.37, meaning that there is no publication bias (Fig. 4).

### Determinants of diabetic dyslipidemia

#### Female

**Fig. 5:**
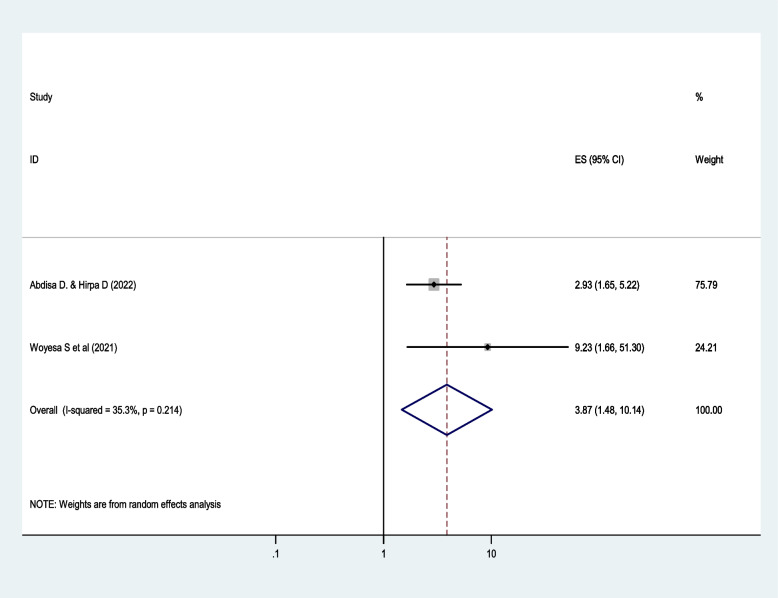
The effect of sex on dyslipidemia among diabetic patients

Being female is found to be the determinant factor of dyslipidemia among people with diabetes. Female diabetic patients are 3.9 times more likely to develop dyslipidemia compared with their male counterparts (*AOR*: 3.9, 95% *CI*: 1.5, 10.1; *I*^2^ = 35.3%, *p* = 0.21) using a random effect model (Fig. 5). As shown in the forest plot, the variation between studies is 35.3%, indicating there is minimal heterogeneity. Regarding publication bias, Begg and Mazumdar’s test for rank correlation gave a *p*-value of 0.32, indicating no evidence of publication bias.

#### Physical inactivity

**Fig. 6:**
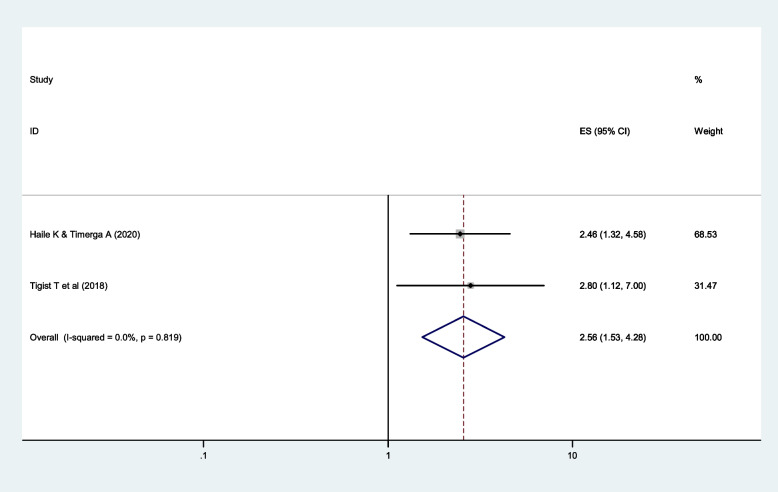
The effect of physical inactivity on dyslipidemia among diabetic patients

Physical inactivity is found to be the determinant factor of dyslipidemia among people with diabetes. Diabetic patients who do not do physical activity were 2.6 times more likely to develop dyslipidemia compared to those people with diabetes who do physical activity (*AOR*: 2.6, 95% *CI*: 1.5, 4.3; *I*^2^ = 0.0%, *p* = 0.82). The *I*^2^ test statistic is 0.0%, and the *p*-value is 0.82, which indicates there is minimal heterogeneity across the included studies (Fig. 6). On further testing, Begg and Mazumdar’s test rank correlation gave a *p*-value of 1.00, indicating there is no publication bias.

#### Uncontrolled blood glucose

**Fig. 7:**
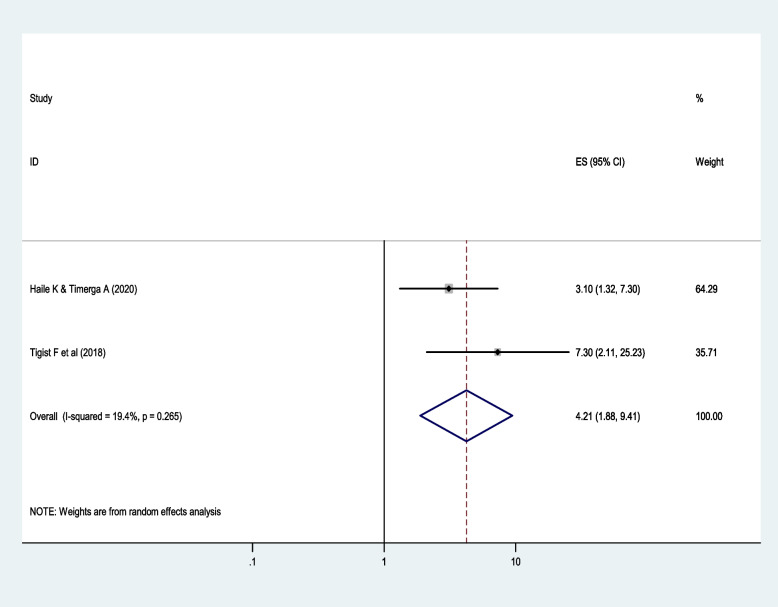
The effect of uncontrolled blood sugar on dyslipidemia among diabetic patients

In this systematic review and meta-analysis, diabetic patients who have uncontrolled blood glucose are at risk for dyslipidemia. People with diabetes who have uncontrolled blood glucose are 4.2 times more likely to develop dyslipidemia compared to those with controlled diabetes (*AOR*: 4.2, 95% *CI*: 1.9, 9.4; *I*^2^ = 19.4%, *p* = 0.27). The *I*^2^ and Cochrane Q statistics *p*-values are 19.4% and 0.27, respectively (Fig. 7). These indicate that there is minimal variation across the studies. Begg and Mazumdar’s test for rank correlation gave a *p*-value of 0.32, indicating there is no evidence of publication bias.

In addition to the above factors, the effects of some sociodemographic, personal-related, and clinical factors on dyslipidemia among people with diabetes in Ethiopia are summarized (Additional File 3).

## Discussion

In this systematic review and meta-analysis, the pooled prevalence of diabetic dyslipidemia was 65.7% (95% *CI*: 57.5, 73.9). The study includes both type 1 and type 2 diabetic patients in Ethiopia. Based on the regions of Ethiopia, the prevalence of diabetic dyslipidemia was 77.5% (95% *CI*: 65.3, 89.6) in SNNP, 64.4% (95% *CI*: 59.2, 69.6) in Amhara, and 63.8% (95% *CI*: 46.0, 81.6) in the Oromia region. The findings of this study are higher than those of the studies conducted in 10 African countries: 52.8% [40] and 25.5% [41]. The possible discrepancy is variation in the study population. In the African studies, the participants were both diabetic and nondiabetic populations and the general population, but the current study focused on diabetic patients.

Based on the lipid profiles, the pooled prevalence of TC, TG, LDL-c, and HDL-c were found to be 34.7% (95% *CI*: 23.3, 46.0), 51.8% (95% *CI*: 45.1, 58.6), 34.4% (95% *CI*: 22.3, 46.6), and 44.2% (95% *CI*: 32.8, 55.7), respectively. This finding is in line with the study in Orlando, Florida, *TC* 30.08% [42]. In this study, the findings of TG and LDL-c are higher than those of the study conducted in Orlando, Florida: *TG* 24.82% and *LDL-c* 18.62%. The variation may be associated with lifestyle modification issues such as physical activity and healthy eating. In addition, socio-economic status plays a vital role in this discrepancy.

In the meta-regression analysis, sample size is the source of the variation for the included studies. The finding of this study is supported by the study conducted in the USA, which dictates that heterogeneity between small studies is greater than between larger studies [43]. This is due to studies with a large sample size, which gives a narrow confidence interval and good precision [44].

In this systematic review and meta-analysis, female diabetic patients are at risk for developing dyslipidemia. The finding is supported by a large survey study in Spain [45] and China [46]. In their reproductive years, the females have a low LDL level, but it rises after menopause. This is associated with female sex hormones such as estrogens, which have a lowering effect specifically on LDL [47, 48]. After the menopause phase, the estrogen level decreased, which in turn increased the LHD levels. Estrogen in women causes a high level of good HDL cholesterol and maintains the bad LDL cholesterol to a lower normal limit during the monthly menstruation cycle [49]. But at a later age, these conditions reverse and lead to a progressive rise in LDL cholesterol levels [50].

People with diabetes who are physically inactive are at risk for dyslipidemia. This is due to the fact that physical inactivity increases visceral fat accumulation, stimulates chronic low-grade systemic inflammation, and leads to insulin resistance and dyslipidemia [51]. Sedentary lifestyles increase the deposit of bad cholesterol. This contributes to endothelial dysfunction and atherosclerosis [52], and in later life, this results in life-threatening cardiovascular complications [14]. Furthermore, the findings of this study showed that uncontrolled blood glucose is the determinant of dyslipidemia among people with diabetes. Glucose and lipid metabolism are linked to each other in many ways, which leads to diabetic dyslipidemia or increased lipid profiles [53]. Evidence has been reported that dyslipidemia is associated with insulin resistance and increased lipid metabolism [54, 55].

This review has the following important limitations: (1) The study included both diabetic patients, such as type 1 and/or type 2, and as a result, it does not clearly show which population type is more affected. Therefore, further study is needed on type 1 or type 2 diabetic patients separately. In the positive aspects, the authors used major databases to search related articles and screened them independently using Rayyan software.

### Implications of the study

The main finding is the considerable, unexplained variation in prevalence. The study highlights the prevalence of dyslipidemia among people with diabetes. The findings of the study provide input to healthcare workers on how to carry out regular lipid profile screening to prevent, detect, and treat lipid profile abnormalities to minimize possible complications. Furthermore, it helps decision-makers and policymakers plan preventive measures before the occurrence of life-threatening cardiovascular complications.

## Conclusion

This review revealed that the prevalence of diabetic dyslipidemia is high among people with diabetes in Ethiopia. Being female, having physical inactivity, and having uncontrolled blood glucose were the predictors of dyslipidemia. In Ethiopia, lipid-lowering agents and antidiabetic treatments have been routinely provided for people with diabetes with comorbid dyslipidemia. Therefore, regular screening of lipid profiles and treatment of hyperglycemia should be strengthened. Furthermore, lifestyle modification interventions such as a healthy diet, regular physical exercise, and adequate blood glucose control need to be encouraged to reduce life-threatening cardiovascular complications. Healthcare providers should also give special attention to women living with diabetes.

### Supplementary Information

Additional file 1: PRISMA checklist.

Additional file 2: Data availability statement. Table 1:  Study characteristics for the age of diabetic patients in Ethiopia.

Additional file 3: Summary of factors associated with dyslipidemia among diabetic patients in Ethiopia.

## Data Availability

The datasets used and/or analyzed during the current study are available from the corresponding author upon reasonable request.

## Abbreviations

CVD: Cardiovascular diseases
DM: Diabetes mellitus
HDL-C: High-density lipoprotein cholesterol
LDL-C: Low-density lipoprotein cholesterol
PRISMA: Preferred Reporting Items of Systematic reviews and Meta-Analysis
PROSPERO: Prospective register of systematic reviews
SNNPR: Southern Nation, Nationalities, and Peoples’ Region
T2DM: Type 2 diabetes mellitus
