# Supplementary material for: Diabetic dyslipidemia and its predictors among people with diabetes in Ethiopia: systematic review and meta-analysis
Source: Syst Rev. 2024 Jul 20;13:190. doi: 10.1186/s13643-024-02593-2 (PMC11264949; doi:10.1186/s13643-024-02593-2)
Supplement: Supplementary file 1 — Additional file 1: PRISMA checklist. [file 13643_2024_2593_MOESM1_ESM.docx]

| **Section and Topic** | **Item #** | **Checklist item** | **Location where item is reported** |
| --- | --- | --- | --- |
| **TITLE** | | |  |
| Title | 1 | **Diabetic dyslipidemia among diabetic patients in Ethiopia: A systematic review and meta-analysis** | 1 |
| **ABSTRACT** | | |  |
| Abstract | 2 | **Introduction:** Dyslipidemia is a lipoprotein disorder promoting the development of atherosclerosis and cardiovascular diseases (CVD). It is the major independent predictor of CVD in diabetic patients, which results in high mortality and morbidity of diabetic patients. In Ethiopia, there are inconsistent findings on dyslipidemia among diabetic patients. The aim of this review study is to estimate the pooled prevalence of dyslipidemia among diabetic patients in Ethiopia. **Method:** The searches were carried out in Medline via Pubmed, Embase, Embsco, Ovid, and other supplementary gateways Google and Google Scholar. The articles were searched and screened by title (ti), abstract (ab), and full-text (ft). The qualities of the eligible studies were checked by Newcastle Ottawa scale. The heterogeneity was detected by eye ball test, Cochrane chi squared test, and I-squared (I^2^). Then subgroup analysis and meta-regression analysis were used to identify the source of the variations. A random effect meta-analysis model was used to estimate the overall pooled prevalence of dyslipidemia. The publication bias was assessed by funnel plot test and/ or Egger’s test (p-value <0.05). The protocol has been registered in an international database, the prospective register of systematic reviews (PROSPERO) with reference number: CRD42023441572  **Result:** A total of 14 articles with 3662 sample size were included in this review study. The pooled prevalence of diabetic dyslipidemia in Ethiopia is fund to be 65.68% (95% CI: 57.45%-73.92%), I^2^= 97%, p-value <0.001.The highest prevalence of dyslipidemia is 77.47% (65.32-89.62) in Southern, Nations, nationalities and People (SNNP) region. In meta-regression analysis, sample size (AOR=4.27, 95%CI: 2.20-6.35, p-value= 0.01) is the source of the variation across the included studies.  **Conclusion:** This reviewed study revealed that the prevalence of dyslipidemia is high. Therefore, aggressive treatment of hyperglycemia, regular screening of lipid profiles, providing lipid lowering agents, and education on life style modification should be implemented to prevent its progression to complication.  **Keywords:** Diabetes Mellitus, dyslipidemia, lipid profile abnormality, Ethiopia. | 2 |
| **INTRODUCTION** | | |  |
| Rationale | 3 | Untreated dyslipidemia results in coronary artery diseases, peripheral vascular diseases, hypertension, heart failure, stroke, renal disease, and other. Even though different intervention strategies such as regular follow-up care, health education, and providing lipid lowering agents have been implemented but still the problem coexists. In Ethiopia, studies reported on the prevalence of diabetic dyslibidemia, but their findings are inconsistent across studies. Therefore, this study aimed to estimate the pooled prevalence of dyslipidemia among diabetic population in Ethiopia. | 3 |
| Objectives | 4 | To determine the pooled prevalence of diabetic dyslipidemia among diabetic patients in Ethiopia |  |
| **METHODS** | | |  |
| Eligibility criteria | 5 | In this review study, articles that fulfill the eligibility criteria: 1) studies on diabetic (type 1 and/or type 2 diabetic) population groups; (2) articles with observational studies such as cross-sectional, cohort, and case-control; (3) articles that report the prevalence/ proportion/incidence of dyslipidemia; (4) studies conducted in Ethiopia; (5) both published and/or unpublished articles; (6) articles published/conducted up-to June 2023; (7) articles written by English; (8) articles conducted at health facility settings were included in the study. Articles with trials, without full text, conference papers, gestational diabetic women, and systematic reviews and meta-analyses were excluded from the study | 6 |
| Information sources | 6 | The searches were carried out in Medline via Pubmed, Embase, Embsco, Ovid, and other supplementary gateways Google and Google Scholar. We used boolean logic operators AND and OR are used to combine the search terms. | 4 |
| Search strategy | 7 | The search strategies were carried out using controlled vocabularies (MeSH terms). The synonym of diabetic nephropathy was identified. Then, the search string was established using the databases. Articles were searched by title (ti), abstract (ab), and/or full-text (ft). Boolean logic operators; “AND” and “OR” were used to combine the search terms. | 4 |
| Selection process | 8 | Eligible research articles were screened by their title (Ti), Abstract (Ab), and full-text. Two reviewers independently reviewed the included articles. | 4 |
| Data collection process | 9 | After the quality assessment of the studies, the data were extracted using the Microsoft excel speed sheet. The extracted data item includes; authors, publication year, study design, region of the study, data collection method, and funding source. Two authors/reviewers were independently extracted the data. The disagreements between the reviewers were resolved by discussion and third reviewer involvement. | 6 |
| Data items | 10a | Articles that have clearly defined effect measures were included | 6 |
|  | 10b | The variables that have direct effect for the occurrence of Diabetic dyslipidemia were identified. | 6 |
| Study risk of bias assessment | 11 | The quality of the study was evaluated using the Newcastle Ottawa quality assessment tool adapted from the cross-sectional, cohort, and case-control studies. To reviewers were independently reviewed the quality of the included studies. The inconveniences between the reviewers were resolved by discussion. A score of ≥7 was considered as high quality score. | 6 |
| Effect measures | 12 | The prevalence, logp, and standard error P or (Sep) were used presentation of results. | 6 |
| Synthesis methods | 13a | Both qualitative synthesis and quantitative analysis were employed. | 4 |
|  | 13b | The prevalence or standard error were used for data presentation or evidence synthesis. | 4 |
|  | 13c | PRISMA flow chart, forest plot, and funnel plot were used to present visually displayed data. | 5 |
|  | 13d | The Cochrane Q statistics and (**I**^2^) were used to assess the heterogeneity status of the included studies. | 7 |
|  | 13e | A random effect model was used for analysis. | 7 |
|  | 13f | The quantitative synthesis was employed | 7 |
| Reporting bias assessment | 14 | Egger’s test was computed to detect publication bias. | 7 |
| Certainty assessment | 15 | The pooled prevalence of the study was estimated. | 7 |
| **RESULTS** | | |  |
| Study selection | 16a | The articles were screened by title, abstract, and full text. Two independent reviewers screened the studies, and any controversy was resolved by discussion. | 8 |
|  | 16b | Studies were excluded because of outcome interest and not full text articles. | 7 |
| Study characteristics | 17 | The search retrieved 1644 original research articles. From this 356, 997 and 105 articles were removed due to duplication, not related to the topic of interest, and population difference respectively. Then after 186 articles retrieved of which 168 articles were removed due to not full-text. About 18 full-text articles were accessed for eligibility of which four article were excluded because of poor quality, reporting without the outcome of interest, and outcome measuremt (**Fig.1**). Finally 14 articles with 3662 diabetic patients were included and retrieved for this review study. From studies, five were from Ahmara [[23-27](#_ENREF_23)], four in SNNP [[28-31](#_ENREF_28)], three in Oromia [[32-34](#_ENREF_32)], one in Tigry [[35](#_ENREF_35)] , and One in Addis Ababa [[36](#_ENREF_36)]. Most of the designs were cross-sectional and the publication year ranged from 2017 to 2022. The data collection methods of majority of the included study were patient interview. The prevalence of diabetic dyslipidemia ranged from 37.5% [[34](#_ENREF_34)] to 91.1% [[31](#_ENREF_31)]. The predominant lipid abnormality was elevated triglyceride. The quality of the included studies had high-quality assessment score with average scores≥7 (**Table 1**). | 7 |
| Risk of bias in studies | 18 | The publication bias was detected by visual inspection of the funnel plot indicating there is a symmetrical distribution of articles and the Egger’s test p-value is 0.37 meaning that there is no publication bias (**Fig.4**). | 8 |
| Results of individual studies | 19 | The result of individual studies are pooled to estimate the overall one. | 9 |
| Results of syntheses | 20a | The risk of bias was assed using Egger’s test. | 8 |
|  | 20b | **Prevalence of diabetic dyslipidemia**  The pooled prevalence of diabetic dyslipidemia in Ethiopia is fund to be 65.68% (95% CI: 57.45%-73.92%), I^2^= 97%, p-value <0.001) (**Fig**. **2**). | 11 |
|  | 20c | **Heterogeneity test**  As shown in the forest plot (**Fig. 2**) the I^2^=97.0% and the Cochrane Q statistic p-value< 0.001 indicating there is a considerable variation across the studies. | 12 |
|  | 20d | **Subgroup analysis**  From the included studies the pooled prevalence of dyslipidemia among diabetic patients were 77.47% (65.32-89.62) in SNNP, and 64.37%(59.15-69.58) in Amhara, and 63.76%(45.95-81.55) in the Oromia regions(**Fig. 3**). | 10 |
| Reporting biases | 21 | **Meta-regression**  To identify the factors attributed to the variations across the included studies; sample size and publication years were used. In this meta-regression analysis sample size was found to be the source of variation (AOR=4.27, 95%CI: 2.20-6.35, p-value= 0.01) (**Table** **2**). | 12 |
| Certainty of evidence | 22 | In this systematic and meta-analysis study, co-morbid HTN, poor glycemic control, and longer duration of diabetes illness were found to be the determinant factors of DR. | 15 |
| **DISCUSSION** | | |  |
| Discussion | 23a | In this systematic review and meta-analysis study, the pooled prevalence of diabetic dyslipidemia is 65.68% (95% CI: 57.45%-73.92%). Based on region of Ethiopia, the prevalence of dyslipidemia among diabetic patients was 77.47% (65.32-89.62) in SNNP, and 64.37 %(59.15-69.58) in Amhara, and 63.76%(45.95-81.55) in the Oromia region. The finding of the study is higher than the study conducted in Orlando Florida TC 30.08%, LDL 18.62%, and TG 24.82% [[37](#_ENREF_37)]. This is due to the time variation between the current and the former study. This study focused on the overall prevalence dyslipidemia, whereas the latter one focused on segments of lipid profiles.  In the meta-regression analysis sample size is the source of the variation for the included studies. This is due to when the sample size is to small, the bias from sampling will increase the frequency sampling[[38](#_ENREF_38)]. Larger sample sizes are required in order to attain a desired normal power [[39](#_ENREF_39)]. Additionally, the variation could be small sample size has small effect size this can lead to the possibility of the variation. | 13 |
|  | 23b | This review study has the following important limitations. 1) The study population is diabetic patients (both type 1 and 2 diabetic patients; 2) the design of the study is observational; 3) The language of the included articles were English; 4) the review focused on Ethiopian studies, and 5) the study focused on prevalence of dyslipidemia, but not determinant factors of dyslipidemia among diabetic patients. Therefore, further study is needed; 1) on type 1 or Type 2 diabetic patients separately, 2) incorporating determinant factors of dyslipidemia; and 3) including articles in Africa and/or in global diabetic population. In the positive aspects the Authors used major databases to search related articles. The authors screened blindly using Ryyan software. | 14 |
|  | 23c | The study has an implication for policymakers and clinicians to plan and implement possible interventions to prevent the occurrence and severe outcome of diabetic dyslipidemia. | 14 |
|  | 23d | The finding of this review study revealed that the prevalence of dyslipidemia is high. Therefore, aggressive treatment of hyperglycemia, regular screening of lipid profiles, providing lipid lowering agents, and education on life style modification should be implemented as a public health priority. | 15 |
| **OTHER INFORMATION** | | |  |
| Registration and protocol | 24a | The protocol has been registered in an international database, the prospective register of systematic reviews (PROSPERO) with reference number: CRD42023441572. |  |
|  | 24b | The review protocol can be accessed via online databases. |  |
|  | 24c | Further amendments may /not needed. |  |
| Support | 25 | The Authors did not receive any fund for this particular study. |  |
| Competing interests | 26 | There is no competing of interest. |  |
| Availability of data, code and other materials | 27 | The data extracted were analysed and included in the result. |  |

*From:* Page MJ, McKenzie JE, Bossuyt PM, Boutron I, Hoffmann TC, Mulrow CD, et al. The PRISMA 2020 statement: an updated guideline for reporting systematic reviews. BMJ 2021;372:n71.doi: 10.1136/bmj.n71

For more information, visit:<http://www.prisma-statement.org/>
