# Supplementary material for: Diabetic dyslipidemia and its predictors among people with diabetes in Ethiopia: systematic review and meta-analysis
Source: Syst Rev. 2024 Jul 20;13:190. doi: 10.1186/s13643-024-02593-2 (PMC11264949; doi:10.1186/s13643-024-02593-2)
Supplement: Supplementary file 2 — Additional file 2: Data availability statement. Table 1: Study characteristics for the age of diabetic patients in Ethiopia. [file 13643_2024_2593_MOESM2_ESM.docx]

**Table 1: Study characteristics for the age of diabetic patients in Ethiopia**

| **Authors** | **year** | **population** | **study**  **design** | **Region** | **data**  **collection** | **sample size** | **prevalence** | **Lipid profile** | **Funding source** | **quality score** |
| --- | --- | --- | --- | --- | --- | --- | --- | --- | --- | --- |
| Abdissa D & Hirpa [[32](#_ENREF_32)] | 2022 | DM | Cross-sectional | Oromia | Interview | 390 | 81.5 | Elevated TGC | Not reported | 7.5 |
| Bekele S, et al. [[28](#_ENREF_28)] | 2017 | DM | Cross-sectional | SNNPR | Interview | 224 | 65.6 | Elevated LDL-C | Not reported | 8 |
| Haile K & Timerga A[[33](#_ENREF_33)] | 2020 | T2DM | Cross-sectional | Oromia | Interview | 248 | 68.1 | Elevated HDL-C | JU | 9 |
| Kebede WM, et al. [[23](#_ENREF_23)] | 2021 | T2DM | Cross-sectional | Amhara | interview | 347 | 59 | Elevated HTG | Nil | 8.5 |
| Woyesa S, et al. [[34](#_ENREF_34)] | 2021 | DM | Cross-sectional | Oromia | Interview | 48 | 37.5 | Hypercholestrolemia | JU | 7 |
| Wuhib M, et al. [[25](#_ENREF_25)] | 2021 | DM | Cross-sectional | Amhara | interview lab test | 250 | 68 | Elevated TG | Not reported | 9 |
| Fikremariam T & Reddy PP[[26](#_ENREF_26)] | 2020 | DM | Cross-sectional | Amhara | Interview  Lab test | 112 | 69.6 | Elevated LDL | BDR | 7.5 |
| Biadgo B, et al.[[24](#_ENREF_24)] | 2018 | T2dm | Cross-sectional | Amhara | Interview , lab test | 159 | 56.6 | Eleveted TG | Nil | 8.5 |
| Birarra MK & Gelaye DA[[27](#_ENREF_27)] | 2018 | T2DM | Cross-sectional | Amhara | Interview, lab test | 256 | 68.8 | Eleveted TG | Not reported | 8 |
| Woyesa SB, et al.[[29](#_ENREF_29)] | 2017 | T2DM | Cross-sectional | SNNP | Interview, lab test | 319 | 70.4 | Elevated TG | JU | 8.5 |
| Gebremeskel GG, et al.[[35](#_ENREF_35)] | 2019 | T2DM | Cross-sectional | Tigray | Interview,  record review | 419 | 45.1 | Eleveted TG | nil | 7 |
| Abebe G et al. [[30](#_ENREF_30)] | 2022 | T2DM | Case-control | SNNPR | Interview. Record review | 204 | 82.18 | Elevated TG | AMU | 9 |
| wube BT, et al. [[31](#_ENREF_31)] | 2020 | T2DM | Cross-sectional | SNNPR | Interview | 314 | 91.1 | Elevated TG | Not reported | 8.5 |
| Birle M, et al.[[36](#_ENREF_36)] | 2019 | T2DM | Cross-sectional | AA | Interview, lab test | 372 | 51.34 | Elevated TG | AMU | 7.5 |

**Notes:- AA**: Addis Ababa; **AMU**: Arba Minch University; **BDU**: Bahir Dar University; **DM**: Diabetes Mellitus; Jimma Universit; **T2DM**: Type 2 Diabetes Mellitus; **SNNPR**: Southern Nation , Nationalities and people region
