# Supplementary material for: Diabetic dyslipidemia and its predictors among people with diabetes in Ethiopia: systematic review and meta-analysis
Source: Syst Rev. 2024 Jul 20;13:190. doi: 10.1186/s13643-024-02593-2 (PMC11264949; doi:10.1186/s13643-024-02593-2)
Supplement: Supplementary file 3 — Additional file 3: Summary of factors associated with dyslipidemia among diabetic patients in Ethiopia. [file 13643_2024_2593_MOESM3_ESM.docx]

**Additional file 3**: Summary of factors associated with dyslipidemia among diabetic patients in Ethiopia

| **Authors** | **year of publication** | **Region s** | **Exposure variables** | **Effect size** | **reference** | **Comments** |
| --- | --- | --- | --- | --- | --- | --- |
|  |  |  | **Socio- demographic factors** | | |  |
| Abdisa D. & Hirpa D[[35](#_ENREF_35)] | 2022 | Oromia | Age > 50years | AOR = 3.24 | < 30years | Participants in their 5^th^ decade (above 50years) were 3.24 times more likely to develop dyslipidemia compared to patients younger than 30 years |
| Haile K & Timerga A[[36](#_ENREF_36)] | 2020 | Oromia | Age ≥ 30 years | AOR = 3.9  P = 0.01 | < 30years | Older T2DM patients (age = 30 years) were nearly 4 times more likely to develop dyslipidemia than lower age groups. |
| Bekele S et al[[31](#_ENREF_31)] | 2017 | SNNPR | Age > 30years | AOR = 8.0 | < 30years | Older diabetic patients were 8 times more likely to develop dyslipidemia (adjusted odd ratio than lower age-groups. |
|  |  |  | **Personal related factors** | | |  |
| Abdisa D. & Hirpa D[[35](#_ENREF_35)] | 2022 | Oromia | Alcohol consumption (yes) | AOR = 2.68  P = 0.006 | No | Those participants who have history of alcohol consumption were 2.68 times more likely to develop dyslipidemia than counterpart |
| Fikremariam T et al[[29](#_ENREF_29)] | 2020 | Amhara | Fat diet product intake(yes) | AOR = 3.33  P = 0.037 | No | Diabetic patients who eat fat diet were 3.33 times at risk to develop dyslipidemia. |
|  |  |  | **Clinical related factors** | | |  |
| Bekele S et al[[31](#_ENREF_31)] | 2017 | SNNPR | Longer duration of DM  6-10 years  >10years | AOR = 8.531  P = 0.001  AOR = 13.816  P = 0.001 | < 6 years | Diabetic patients with between 6–10 years and >10 years were, 8.5 times and 13.8 times more likely to develop dyslipidemia compared with lower age groups |
|  |  |  | Obese  Overweight | AOR = 12.6  P = 0.009  AOR = 8.09  P = 0.004 | Underweight | Obese and overweight diabetic patients were, respectively, 12.6 times and 8.09 times more likely to develop dyslipidemia compared to other diabetic patients. |
|  |  |  | Hypertension | AOR = 1.331  P = 0.016 | No | Diabetic patients with hypertension were 1.331times more likely to develop dyslipidemia. |
| Haile K & Timerga A[[36](#_ENREF_36)] | 2020 | Oromia | Hypertension  (Yes) | AOR = 2.65  P = 0.01 | No | Hypertensive T2DM patients were higher odds of dyslipidemia compared to non-hypertensive patients. |
|  |  |  | BMI | AOR = 12.605  P = 0.004 | Underweight | Obese diabetic patients were 12.6 times more likely to develop dyslipidemia |
| Woyesa S et al[[37](#_ENREF_37)] | 2021 | Oromia | Systolic hypertension | AOR = 3.92  P = 0.048 | Normal | Diabetic patients with high systolic hypertension nearly four times more likely to develop dyslipidemia |
|  |  |  | Diastolic hypertension | AOR = 13.04  P = 0.011 | Normal | Diabetic patients with high diastolic hypertension nearly 13 times more likely to develop dislipidemia |
| Fikremariam T et al[[29](#_ENREF_29)] | 2020 | Amhara | BMI (Abnormal) | AOR = 5.02  P = 0.003 | Normal | Diabetic patients with abnormal BMI were 5 times more likely to develop dyslipidemia compared with normal BMI. |

**Notes: AOR -** Adjusted odds ratio**, BMI -** Body mass index, **P-** P value**, SNNPR** - Southern Nation, Nationalities and people of region, **T2DM-** Type 2 diabetes mellitus
